# Effects of selenoprotein S on oxidative injury in human endothelial cells

**DOI:** 10.1186/1479-5876-11-287

**Published:** 2013-11-14

**Authors:** Yin Zhao, Hua Li, Li-li Men, Rong-chong Huang, Hai-cheng Zhou, Qian Xing, Jun-jie Yao, Chun-hong Shi, Jian-ling Du

**Affiliations:** 1 Department of Endocrinology, The First Affiliated Hospital of Dalian Medical University, Dalian 116011, Liaoning, China; 2 Department of Pharmacology, College of Pharmacy, Dalian Medical University, Dalian 116044, Liaoning, China

**Keywords:** Endothelial cell dysfunction, Oxidation, Selenoprotein S, Caveolin-1, Protein kinase Cα

## Abstract

**Background:**

Selenoprotein S (SelS) is an important endoplasmic reticulum and plasma membrane-located selenoprotein implicated in inflammatory responses and insulin resistance. However, the effects of SelS on endothelial cells (ECs) have not been reported. In the present study, the role of SelS in oxidative stress and the underlying mechanism were investigated in human ECs.

**Methods:**

A SelS over-expression plasmid (pc-SelS) and a SelS-siRNA plasmid were transfected into human umbilical vein endothelial cells (American Type Culture Collection, USA). The cells were divided into four groups: control, SelS over-expression (transfected with pc-SelS), vector control, and SelS knockdown (transfected with siRNA-SelS). After treating the cells with H_2_O_2_, the effects of oxidative stress and the expression of caveolin-1 (Cav-1) and protein kinase Cα (PKCα) were investigated.

**Results:**

Following treatment with H_2_O_2_, over-expression of SelS significantly increased cell viability and superoxide dismutase (SOD) activity, and decreased malondialdehyde (MDA) production and Cav-1 gene and protein expression. However, no effects on PKCα were observed. In contrast, knockdown of SelS significantly decreased cell viability, SOD activity, and PKCα gene and protein expression, and increased MDA production and Cav-1 gene and protein expression.

**Conclusions:**

SelS protects ECs from oxidative stress by inhibiting the expression of Cav-1 and PKCα.

## Background

Atherosclerosis (AS) is a major life-threatening disease in modern society. However, the exact mechanism of AS development is not completely understood and effective therapies are still required [1]. It has been shown that endothelial cell dysfunction (ECD) not only triggers but also promotes the development of AS [2]. Moreover, oxidative stress is considered to be a primary event in the pathogenesis of ECD and AS. Therefore, many resources have been directed towards research into endothelial protection strategies against oxidative injury in recent years.

Many selenoproteins, including glutathione peroxidase 1 (GPx1), thioredoxin reductases (TRs), selenoprotein W (SelW) and selenoprotein P (SelP), participate in intracellular redox homeostasis and play antioxidative roles [3-6]. Tanis, the homolog of Selenoprotein S (SelS), was first characterized as a transmembrane protein in the liver of the Israeli sand rat, *Psammomys obesus*[7]. SelS expression was later found in a pancreatic β cell line, endothelial cells (ECs), human adipose tissue and skeletal muscle tissue [8-10]. The expression of SelS is related to inflammation and insulin resistance (IR) in liver and adipose tissue [8,11,12]. High expression of SelS protected the pancreatic β cell line, Min6, from oxidative damage induced by H_2_O_2_[8]. Reduced expression of SelS led to more severe inflammation in a lipopolysaccharide (LPS)-injured hepatic cancer cell line [12], suggesting that SelS may provide a link between IR, inflammation and oxidative stress pathways through its role as an antioxidant. Although the effect of SelS on oxidative stress in ECs has not yet been reported, it is well known that oxidative stress is the common key factor of IR, AS and ECD. Therefore, SelS/Tanis is likely to be an important mechanistic target for the prevention and therapy of AS-ECD.

Caveolae, a special type of lipid raft, are highly expressed in endothelial membranes. Caveolin-1 (Cav-1), the principal marker protein of caveolae, participates in the pathologic processes of inflammation and IR [13,14], leading to ECD. The special scaffold structure of Cav-1 modulates the activity of protein kinase C (PKC) during oxidative stress, while PKC regulates gap junctions through Cav-1-containing caveolae. PKC is recruited into Cav-1-containing lipid rafts in the cell membrane from the cytoplasm to induce the redistribution of PKC and Cav-1 in lipid rafts, suggesting a possible interaction between Cav-1 and PKC [15]. SelS has four potential phosphorylation sites for PKC. SelS is also similar to Cav-1 in that it is a membrane protein highly expressed in ECs. To our knowledge, the interaction between SelS and Cav-1 in the oxidative stress pathway of ECs has not been reported previously. In this study, we investigated whether SelS participates in the oxidative stress pathway in ECs using a gain/loss of SelS function strategy combined with analysis of the expression levels of Cav-1 and PKCα following treatment with H_2_O_2_.

## Methods

### Construction of the SelS over-expression plasmid, pc-SelS

A SelS gene fragment (579 bp) was amplified from human adipose tissue by reverse-transcription polymerase chain reaction (RT-PCR). Specific primers were designed according to the nucleotide sequence of human SelS [GeneBank: NM-018445]. The sense primer sequence was 5′-CCGCTCGAGATGGAACGCCAAGAGGAGTCTCTG-3′, and the antisense primer sequence was 5′-CCGGAATTCGCCTCATCCGCCAGATGACGGGCC-3′. Restriction enzyme sites were introduced into the sense primer (XhoI at the 5′ end) and antisense primer (EcoRI at the 3′ end) to enable subcloning of the amplified fragment. The PCR product was cloned into the pMD18-T Simple Vector and then digested with XhoI and EcoRI. The digested SelS DNA fragment was purified and subcloned into the pcDNA3.1 eukaryotic expression vector to generate the pcDNA3.1-SelS recombinant plasmid (pc-SelS). After confirming the DNA sequence, pc-SelS was transiently transfected into human umbilical vein endothelial cells (HUVECs) via liposomes.

### Construction of the SelS low expression plasmid, siRNA-SelS

Three interfering target sequences and one negative control (HK) were designed according to the human SelS gene sequence [GeneBank: NM-018445] using online software provided by Wuhan Jing Contest Company and synthesized into double-strand DNA templates. The sequences were as follows: SelS1, 5′-GCAUCCUUCUCUACGUGUUCAAGACGCACGUAGAGAAGGAUGCUU-3′; SelS2, 5′-CUGUGGAACCUGAUGUUUUCAAGACGAACAUCAGGUUCCCAAGUU-3′; SelS3, 5′-AGCAGCUGCUCGACUGAUUCAAGACGUCAGUCGAGCAGCUGCUUU-3′; HK, 5′-GACUUCAUAAGGCGCAUUUCAAGACGAUGCGCCUUAUGAAGUCUU-3′. After cloning into the pcDNA3.1 eukaryotic expression vector and sequencing, the correct clones were transformed into *E. coli* DH5α. Kanamycin-resistant clones were expanded and three interfering plasmids for siRNA-SelS were sequenced before transfection into HUVECs.

The conditions for plasmid transfection were initially optimized. Briefly, HUVECs were seeded in six-well plates and cultured in minimum essential medium supplemented with 10% fetal bovine serum (Invitrogen Corp, USA) for 24 hours. When the cells had reached approximately 90% confluence, transfection was performed in serum-free medium using Lipofectamine 2000 (Invitrogen Corp), with different ratios of plasmid to Lipofectamine. According to the transfection efficiency under a fluorescence microscope, a 1:1.5 ratio of plasmid (μg) to liposome (μl) was selected for the experiments.

**Figure 1:**
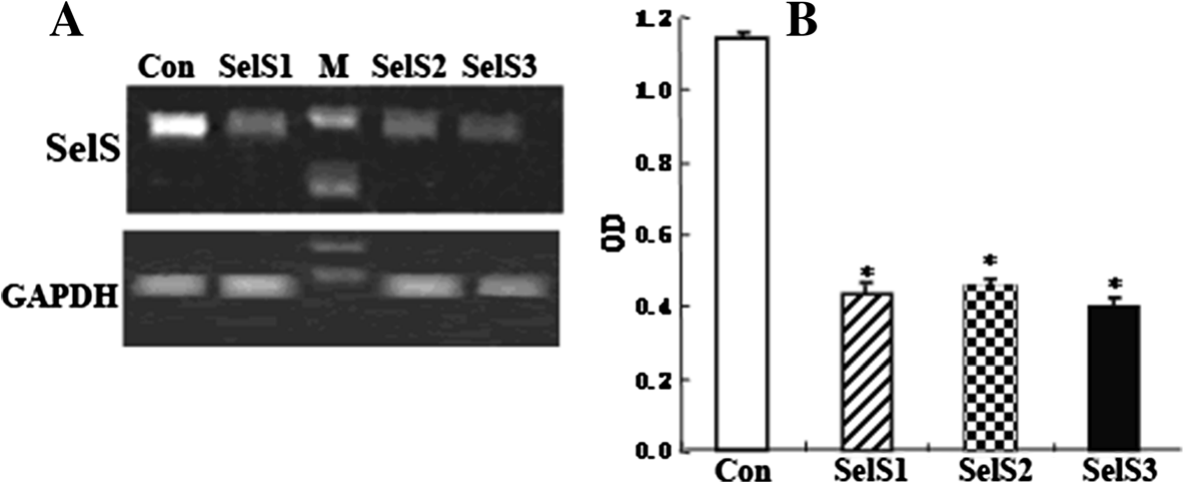
**RT-PCR analysis of SelS gene expression after transfection of siRNA-SelS into HUVEC cells. A**. RT-PCR analysis of SelS mRNA in cell lysates. **B**. The level of SelS mRNA expression presented as a ratio to GAPDH following densitometric analysis of the RT-PCR products. M: marker; Con: control group; SelS1, SelS2, SelS3: siRNA-SelS1, 2 and 3 plasmids, respectively. *P < 0.01 compared with the control group.

The interfering effects of three siRNA-SelS plasmids on gene expression were investigated. Briefly, 4 hours after transfection, cells were transferred into normal medium for 24 hours before being harvested. Gene expression was analyzed by RT-PCR. Finally, one siRNA-SelS plasmid was chosen for the following experiments according to its interfering effects (Figure 1).

### Expression of pc-SelS and siRNA-SelS in HUVECs

HUVECs were divided into the following four groups: normal control group, vector control group, pc-SelS group (SelS over-expression group transfected with pc-SelS), and siRNA-SelS group (SelS low expression group transfected with siRNA-SelS). The cells were harvested 24 hours after transfection. Gene expression was analyzed using real-time PCR and protein expression was analyzed by western blotting with a rabbit anti-human SelS polyclonal antibody (prepared by Wuhan Jing Contest Company, China).

### Effects of SelS on H_2_O_2_-injured HUVECs

#### Analysis of cell viability using the MTT assay

HUVECs were divided into the four groups described above (normal control group, vector control group, pc-SelS group, siRNA-SelS group). Twenty-four hours after transfection, all groups were treated with different concentrations of H_2_O_2_ (0, 400, 600, 800 or 1000 μmol/L) for 2 hours, and the HUVECs were then washed to remove the H_2_O_2_ prior to addition of 3-(4,5-dimethylthiazol-2-yl)-2,5-diphenyl tetrazolium bromide (MTT). Then, the HUVECs were incubated with MTT (0.5 mg/mL) at 37°C for 4 hours. The solution was then removed and the formazan salts were dissolved with dimethyl sulphoxide, and the absorbance at 570 nm of the resulting solution was measured.

#### Determination of MDA production and superoxide dismutase (SOD) activity

The MDA level and SOD activity were analyzed using specific reagents according to the protocols provided by the manufacturer (Nanjing Jiancheng Bioengineering Institute, China). Briefly, thiobarbituric acid was used as substrate for the detection of MDA, and the xanthine oxidase method was used for the detection of SOD activity.

### Real-time PCR

After treatment with 800 μmol/L H_2_O_2_ for 24 hours, the cells in all groups were harvested and total RNAs were extracted using Trizol reagent (Invitrogen, USA). The levels of the mRNAs for Cav-1 and PKCα were examined by real-time PCR analysis. The sequences of the specific primers for Cav-1 and PKCα used for real-time PCR were as follows. Cav-1: sense primer, 5′-AACCTCCTCACAGTTTTCATCCA-3′, antisense primer, 5′-GTCGTACACTTGCTTCTCGCTCA-3′; PKCα: sense primer, 5′-CCTTCAGACAAAGACCGACGACT-3′, antisense primer, 5′-CTTCATCAGCTCCGAAACTCCAA-3′; GAPDH: sense primer, 5′-CGACACCCACTCCTCCACCTTTG-3′, antisense primer, 5′-TCCACCACCCTGTTGCTGTAGCC-3′. The SYBR Green PCR Master Mix kit (Takara Biotechnology Co. Ltd.) was used according to the manufacturer’s protocol. The real-time PCR reactions were performed using an Applied Biosystems 7500 Real-time PCR System (Life Technologies, USA). The PCR mix was first denatured at 95°C for 10 seconds, followed by 40 cycles of 95°C for 5 s, 59°C for 10 seconds and 72°C for 10 seconds. The data presented are from three independent experiments.

### Western blotting

After treatment with 800 μmol/L H_2_O_2_ for 24 hours, the cells in all groups were harvested and lysed for the extraction of total proteins. The protein content was determined using the Bradford assay. Briefly, 30 μg protein samples were separated on sodium dodecyl sulfate polyacrylamide gels and transferred to polyvinylidene difluoride membranes (Millipore). A rabbit anti-human Cav-1 antibody (1:750, Santa Cruz, USA), a horseradish peroxidase (HRP)-labeled goat anti-rabbit secondary antibody (1:6000, Santa Cruz, USA), a mouse anti-human PKC antibody (1:200, Santa Cruz, USA) and a HRP-labeled goat anti-mouse secondary antibody (1:2000, Santa Cruz, USA) were used separately. The signals were detected using an enhanced chemiluminescence method (Hybond ECL, Amersham Bioscience, USA). The protein bands were recorded and analyzed using visionworksLS software (UVP Inc., USA). The expression levels of Cav-1 and PKCα protein were expressed as the density ratio divided by the density of β-actin.

### Statistical analyses

Data are presented as mean ± S.D. Statistical analyses were performed using a paired *t*-test and one-way analysis of variance followed by the LSD method with SPSS version 14.0 software. P values of <0.05 were considered to be statistically significant.

## Results

### Construction of the SelS over-expression and interference plasmids

#### Construction of the SelS over-expression plasmid, pc-SelS

The amplified SelS cDNA sequence was identical to the sequence in Genebank (NM-018445). The eukaryotic expression plasmid, pc-SelS, was further verified by restriction endonuclease digestion and DNA sequencing.

#### Construction and screening of the SelS interference plasmids

The three siRNA-SelS plasmids were verified by sequencing. A plasmid to liposome ratio of 1:1.5 was chosen for liposome transfection.

Gene expression analysis showed that the expression of SelS was significantly inhibited in all three siRNA-SelS transfection groups (P < 0.01), and siRNA-SelS1 was selected for the following experiment.

#### Expression of siRNA-SelS and pc-SelS in HUVECs

**Figure 2:**
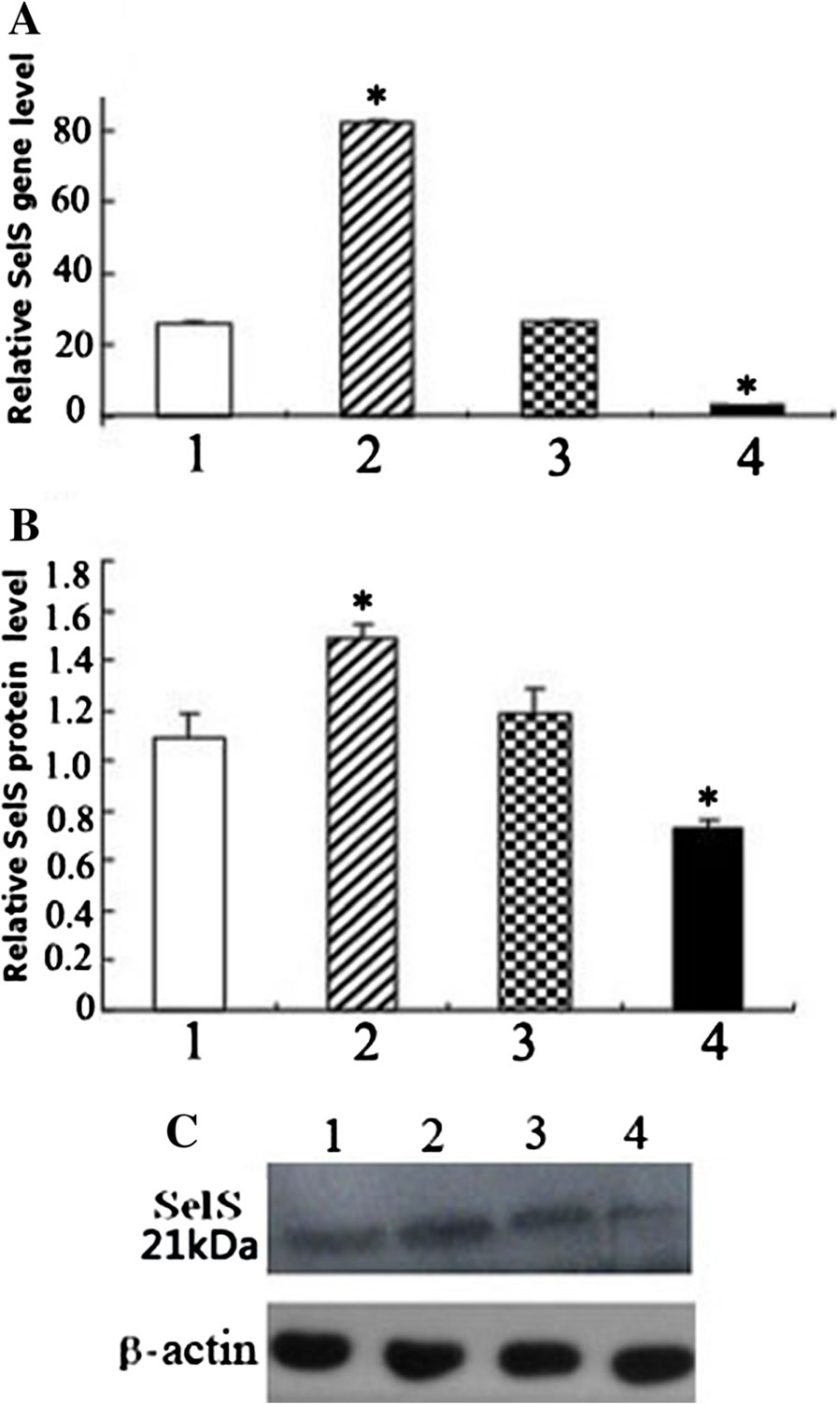
**Analysis of SelS expression in transfected HUVECs. A**. Level of SelS mRNA expression presented as a ratio to GAPDH following real-time PCR analysis. **B**. Level of SelS protein expression presented as a ratio to β-actin following densitometric analysis of western blots. **C**. Western blot analysis of SelS. 1, 2, 3, 4: control group, pc-SelS group, vector control group and siRNA-SelS group, respectively. *P < 0.01 compared with control group.

Real-time PCR analysis showed that SelS gene expression in both the pc-SelS over-expression group (82.51 ± 0.42, P < 0.01 *versus* vector control group) and the siRNA-SelS low expression group (2.67 ± 0.16, P < 0.01 *versus* vector control group) was significantly different from the vector control group (26.54 ± 0.35). Additionally, expression of the SelS mRNA in the low expression group was knocked down by approximately 90% (Figure 2A and B).

Western blot analysis showed similar results, and the expression of SelS protein in the siRNA-SelS group was decreased by approximately 30% (Figure 2C).

### Effects of SelS on H_2_O_2_-induced EC injury

#### Effects of SelS on cell viability

**Figure 3:**
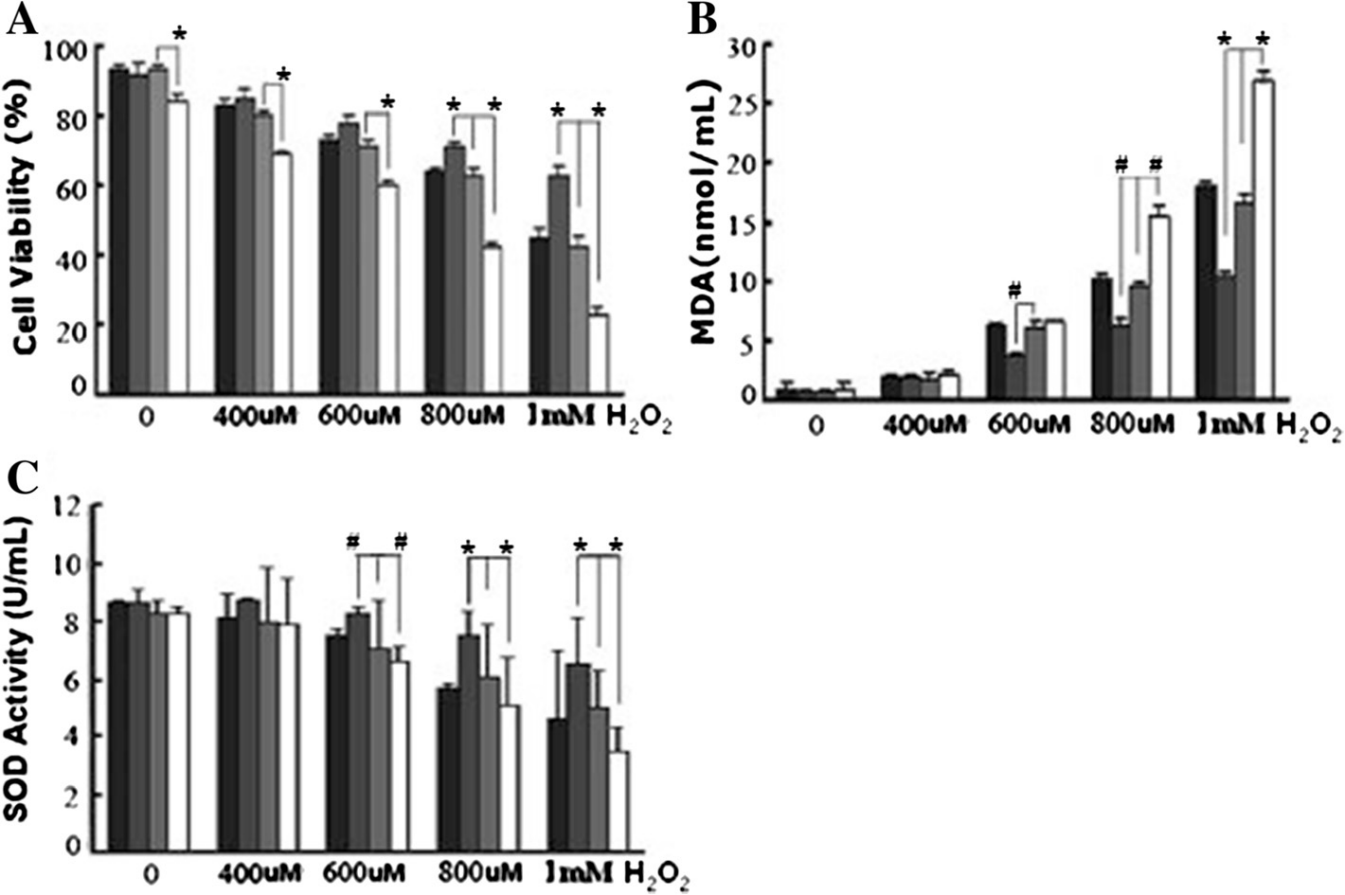
**Effects of SelS on H**_**2**_**O**_**2**_**-induced EC injury. A**. Effects of SelS on cell viability. **B**. Effects of SelS on MDA production. **C**. Effects of SelS on SOD activity. The black, dark grey, grey and white columns indicate the control group, pc-SelS group, vector control group and siRNA-SelS group, respectively. *P < 0.05, ^#^P < 0.01 compared with vector control group. n = 3.

All groups of HUVECs showed decreased cell viability after exposure to different concentrations of H_2_O_2_ for 6 hours. Cell viability in the siRNA-SelS group was significantly lower than that in the other groups (P < 0.01), while cell viability in the SelS over-expression group was significantly higher than that in the other groups (P < 0.01) following stimulation with H_2_O_2_ at concentrations of 800 and 1000 μmol/L (Figure 3A).

#### Effects of SelS on MDA production

The MDA content in the cell medium increased when the concentration of H_2_O_2_ was increased. The MDA content in the siRNA-SelS group was significantly higher than that in the SelS over-expression group, the normal control group and the vector transfection group (treated with H_2_O_2_ at concentrations of 600, 800 and 1000 μmol/L, respectively). The MDA content in the SelS over-expression group was significantly lower than in the other groups at these three concentrations of H_2_O_2_ (Figure 3B).

#### Effects of SelS on SOD activity

SOD activity in the siRNA-SelS group was significantly decreased, while SOD activity in the SelS over-expression group was significantly increased, by H_2_O_2_ treatment compared with the other groups (P < 0.01) (Figure 3C).

### Effects of SelS on Cav-1 expression

**Figure 4:**
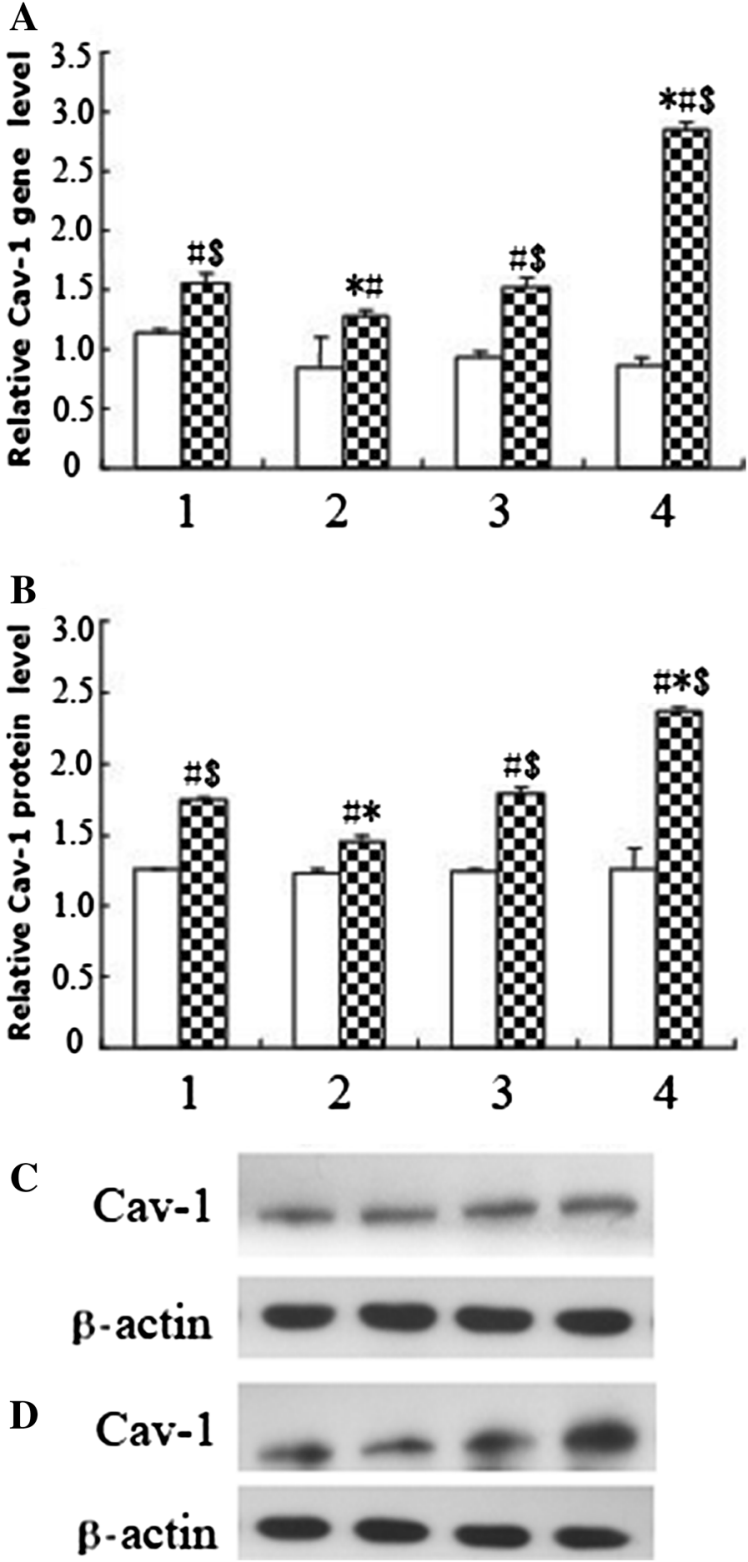
**Effects of SelS on the expression of Cav-1 in ECs stimulated with or without H**_**2**_**O**_**2**_**. A**. The level of Cav-1 gene expression presented as a ratio to GAPDH following real-time PCR analysis. **B**. The level of Cav-1 protein expression presented as a ratio to β-actin following densitometric analysis of western blots. **C**. Western blot analysis of Cav-1 expression in HUVECs not stimulated with H_2_O_2_. **D**. Western blot analysis of Cav-1 expression in HUVECs stimulated with 800 μmol/L H_2_O_2_. White column: no H_2_O_2_ stimulation; shaded column: stimulation with 800 μmol/L H_2_O_2_. 1, 2, 3, 4: control group, pc-SelS group, vector control group and siRNA-SelS group, respectively. ^#^P < 0.01 compared with the corresponding control groups not stimulated with H_2_O_2_; *P < 0.01 compared with the vector control group; ^$^P < 0.01 compared with the SelS over-expression group. n = 3.

Cav-1 gene and protein expression were significantly increased by treatment with 800 μmol/L H_2_O_2_ compared with the corresponding control groups not treated with H_2_O_2_ (P < 0.01). The levels of Cav-1 gene and protein were significantly decreased in the SelS over-expression group, and significantly increased in the siRNA-SelS group, when compared with the vector control groups after H_2_O_2_ treatment (P < 0.01) (Figure 4).

### Effects of SelS on PKCα expression

**Figure 5:**
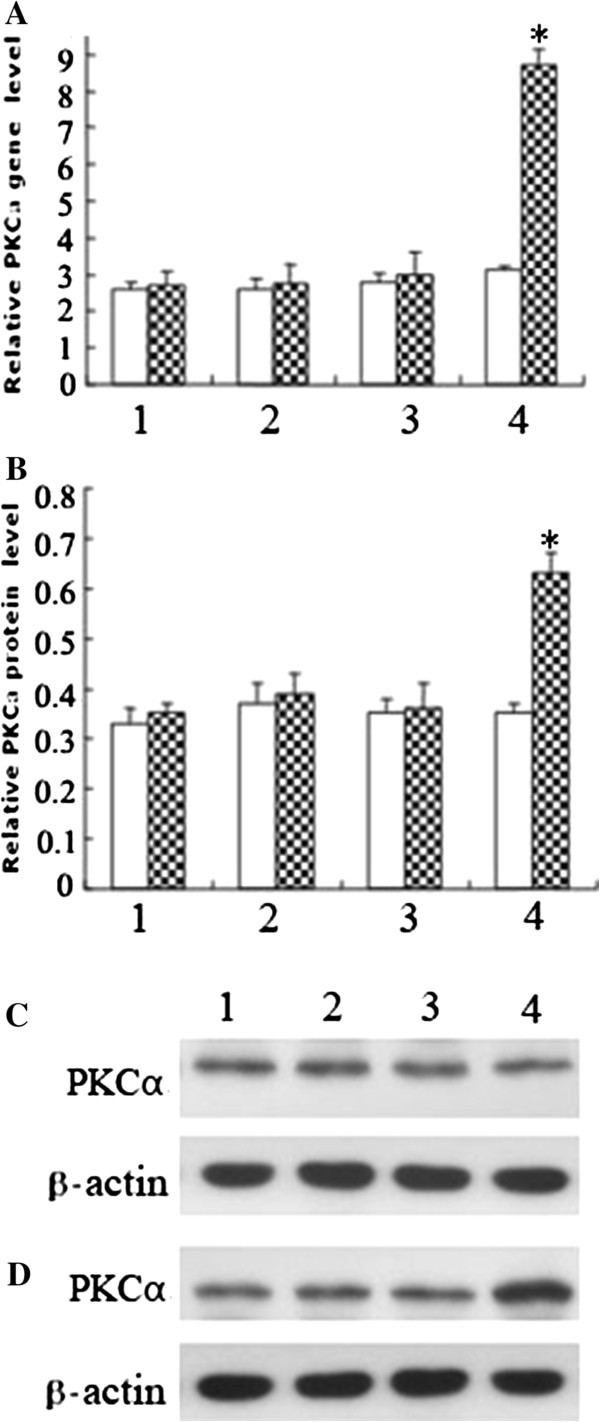
**Effects of SelS on the expression of PKCα in ECs stimulated with or without H**_**2**_**O**_**2**_**. A**. The level of PKCα gene expression presented as a ratio to GAPDH following real-time PCR analysis. **B**. The level of PKCα protein expression presented as a ratio to β-actin following densitometric analysis of western blots. **C**. Western blot analysis of PKCα expression in HUVECs not stimulated with H_2_O_2_. **D**. Western blot analysis of PKCα expression in HUVECs stimulated with 800 μmol/L H_2_O_2_. White column: no H_2_O_2_ stimulation; shaded column: stimulation with 800 μmol/L H_2_O_2_. 1, 2, 3, 4: control group, pc-SelS group, vector control group and siRNA-SelS group, respectively. *P < 0.01 compared with the vector control group. n = 3.

PKCα gene and protein expression were significantly increased in the siRNA-SelS group treated with 800 μmol/L H_2_O_2_ compared with the corresponding groups treated with or without H_2_O_2_ (P < 0.01). No significant changes were observed in the other groups (P > 0.05) (Figure 5).

## Discussion

The novel selenoprotein, Tanis, was first identified in *P. obesus*, an animal model of type 2 diabetes and the metabolic syndrome. SelS, the human homolog of Tanis, was later found to be expressed in a variety of tissues as an important endoplasmic reticulum (ER) and plasma membrane-located selenoprotein.

SelS has been reported to have multiple biological activities. SelS promoted the reverse translocation of unfolded and misfolded proteins to the cytoplasm from the ER space for degradation after ubiquitination to maintain ER homeostasis [16]. Moreover, the SelS gene locates within a region of chromosome 15q26.3, which also contains many inflammation-related loci. It was shown that proinflammatory cytokines activated the transcription of SelS, and up-regulation of SelS inhibited the production of cytokines through a feedback loop [7,17]. SelS also protected Min6 islet cells from oxidative stress-induced toxicity [4]. However, the role of SelS in ECs has not yet been reported. Therefore, in the present study, the antioxidative effects of SelS in ECs were investigated using a gain/loss of function approach.

The results of the present study demonstrated that increased expression of SelS protected HUVECs from H_2_O_2_ damage by inducing increased cell viability and SOD activity, and decreased MDA production. Interfering with the SelS gene significantly decreased the antioxidative activity of HUVECs treated with H_2_O_2_, as shown by decreased cell viability and SOD activity, and increased MDA production. Our results are consistent with those of Zeng et al., who reported that interfering with SelS gene expression exacerbated LPS-induced inflammation injury in a liver cancer cell line [8]. Moreover, the present study demonstrated that expression of Cav-1 in ECs was increased by stimulation with H_2_O_2_, while the increase in Cav-1 in the SelS over-expression group was significantly lower than that in the SelS low expression group. These results suggest that the protective effect of SelS in response to H_2_O_2_-induced damage is related to the down-regulation of Cav-1.

Cav-1, the key structural protein in caveolae, is highly expressed in ECs. However, only a few studies on Cav-1 and ECs have been reported. In diabetic rats, oxidative stress in ECs increased the production of VEGF and then induced the gathering of caveolae and increased expression of Cav-1, leading to capillary hyperpermeability to plasma macromolecules and the intensification of membrane-cytoplasm transportation [18]. Later, the role of Cav-1 in the mechanism of oxidant-induced pulmonary vascular hyperpermeability and edema formation was investigated using a Cav-1(-/-) mouse. This study showed that H_2_O_2_ exposure induced the phosphorylation of Cav-1, which resulted in endothelial barrier disruption [19]. Further research also confirmed the induction of Cav-1 following oxidative stress and the fundamental role of Cav-1 in endothelial barrier function [20]. It also has been shown that caveolae and Cav-1 may play an important role in the mediation of native and modified LDL uptake/efflux and transcytotic trafficking in ECs [21]. These findings suggest a pro-atherogenic role of Cav-1 and caveolae. Our present study has shown that SelS may down-regulate Cav-1 expression to protect ECs from oxidative stress, suggesting that SelS plays an inhibitory role during the early pathophysiologic stages of AS.

PKC has been reported to be involved in the mechanism of antioxidative stress. Numerous findings have revealed that PKC alpha is a mediator in the activation of stress-response kinase. Kuo DY et al. demonstrated that PKC alpha knock-down could reverse the increase of SOD gene expression induced by phenylpropanolamine [22]. They revealed that PKC alpha signaling was an upstream mediator that regulates SOD gene expression, and the increase in SOD induced by PKC alpha can reduce oxidative stress. Makino J et al. showed that expression of SOD in the vascular wall was completely blocked by pre-treatment with GF109203X, an inhibitor of PKC, suggesting a direct effect of PKC on SOD expression [23]. Some selenium-containing compounds showed antioxidative effects by inhibiting PKC via redox-active cysteine residues in the catalytic domain of PKC [24,25]. Rayudu G and his team have shown that oxidative stress can stimulate PKC activity, and the interaction of seleno-compounds with the redox-active cysteine-rich regions in PKC is important for their antioxidative function [25]. The present study showed that the expression levels of PKCα gene and protein were significantly increased in the SelS low expression group, but there was no significant change in the SelS over-expression group. Therefore, SelS may have an inhibitory effect on PKCα under normal conditions, while the decreased expression of SelS relieved the inhibition on PKCα leading to increased PKCα expression and oxidative injury. However, high expression of SelS showed no significant effect on the expression of PKCα, suggesting that other mechanisms may be involved. Also, whether other subtypes of PKC (PKCβ, PKCγ) play roles in the SelS pathway should be investigated further.

## Conclusions

Our study showed that up-regulation of SelS inhibited H_2_O_2_-induced Cav-1 expression, and down-regulation of SelS increased the expression of Cav-1 and PKCα. Therefore, our study demonstrated, for the first time, the protective effect of SelS on oxidation-injured ECs. Furthermore, our results suggest that SelS may inhibit expression of Cav-1 to inhibit PKCα and block downstream activation of the PKC signaling pathway, leading to its antioxidative protection effect in ECs. Further experiments will focus on the mechanism in more detail. Specific inhibitors of Cav-1 and PKCα will be used to demonstrate their roles in the SelS effect when SelS is over-expressed or knocked down. Also, co-precipitation of Cav-1 and SelS/PKCα will be performed. Moreover, SelS has been shown to perform complex biological activities via diverse mechanisms during many pathological processes, including oxidative stress, ER stress and cytokine production. Future studies will also focus on the link between the effects of SelS on oxidative stress, ER stress and inflammation. The *in vivo* effects of SelS transfection and knockdown in mice will also be investigated in order to clarify the role of SelS in the organism, which may be more meaningful for the prevention and therapy of AS-ECD.

## Competing interests

The authors declare that they have no competing interests.

## Authors’ contributions

YZ, HL, QX, JJY and LLM designed and performed the experiments. JLD and HL interpreted the results and drafted the manuscript. CHS and HCZ analyzed the data. RCH contributed reagents and materials. All authors read and approved the final manuscript.
